# Long-Term Golimumab Treatment Persistence and Clinical Outcomes in Axial Spondyloarthritis: A Real-World Cohort from Kazakhstan

**DOI:** 10.3390/jcm15208099

**Published:** 2026-10-19

**Authors:** Kanat Nurgaliyev, Bakytsholpan Issayeva, Nikolay Soroka, Ainur Sadykova, Svetlana Tuleutayeva, Zhansaya Nurgaliyeva, Indira Karibayeva

**Affiliations:** 1 Department of Rheumatology, Kazakh National Medical University Named After S.D. Asfendiyarov, Almaty 050044, Kazakhstan; nurgaliyev.kanat@kaznmu.kz (K.N.); issayeva.b@kaznmu.kz (B.I.); 2 Department of Internal Medicine No. 2, Belarusian State Medical University, 220000 Minsk, Belarus; soroka1949@mail.ru; 3 Department of Infectious and Tropical Diseases, Kazakh National Medical University Named After S.D. Asfendiyarov, Almaty 050044, Kazakhstan; 4 Department of General Medical Practice № 1, Kazakh National Medical University Named After S.D. Asfendiyarov, Almaty 050044, Kazakhstan; tuleutaeva.s@kaznmu.kz; 5 Faculty of Medicine and Health Care, Farabi University, Almaty 050044, Kazakhstan; 6 Department of Health Policy and Community Health, Jiann-Ping Hsu College of Public Health, Georgia Southern University, Statesboro, GA 30460, USA; ik01379@georgiasouthern.edu; 7 Department of Research Management, Joint Stock Company “Research Institute of Cardiology and Internal Diseases”, Almaty 050012, Kazakhstan

**Keywords:** axial spondyloarthritis, ankylosing spondylitis, golimumab, tumor necrosis factor inhibitor, drug retention, treatment persistence, real-world evidence, Kazakhstan

## Abstract

**Background/Objectives**: Long-term real-world evidence on golimumab in axial spondyloarthritis (axSpA) from Central Asia is limited. We evaluated clinical outcomes through week 144, treatment persistence through 36 months, factors associated with discontinuation, and reasons for discontinuation in Kazakhstan. **Methods**: This prospective observational cohort included 113 adults with axSpA enrolled at two rheumatology centers in Almaty in October 2019. All had received golimumab 50 mg subcutaneously every four weeks for at least 12 weeks before enrollment. Time zero for persistence analyses was the enrollment date. Clinical assessments were performed at baseline and weeks 12, 24, 52, 104, and 144. Treatment persistence was estimated using Kaplan–Meier analysis, and factors associated with confirmed discontinuation were explored using Cox regression. Clinical responses were assessed using non-responder imputation (NRI; N = 113). **Results**: During 36 months, 24 patients had confirmed permanent discontinuation; two patients lost to follow-up without confirmed discontinuation were censored. Kaplan–Meier estimated persistence was 89.3% (95% CI, 83.8–95.2%) at 12 months, 79.4% (72.2–87.3%) at 24 months, and 78.5% (71.2–86.5%) at 36 months. Among 88 patients with paired baseline and week 144 assessments, the Bath Ankylosing Spondylitis Disease Activity Index (BASDAI), Ankylosing Spondylitis Disease Activity Score based on erythrocyte sedimentation rate (ASDAS-ESR), Bath Ankylosing Spondylitis Functional Index (BASFI), Bath Ankylosing Spondylitis Metrology Index (BASMI), and Maastricht Ankylosing Spondylitis Enthesitis Score (MASES) improved significantly (all *p* < 0.001). With NRI, 64.6% achieved BASDAI50, 59.3% ASDAS inactive disease, and 54.9% ASDAS major improvement at week 144. Higher Rheumatic Disease Comorbidity Index (RDCI) score (adjusted hazard ratio [HR] 1.312, 95% CI, 1.042–1.653) and functional class III–IV versus I–II (adjusted HR 2.412, 95% CI, 1.037–5.611) were associated with higher discontinuation hazards in exploratory analyses. Loss of effectiveness was the most frequent reason for discontinuation (54.2%). Adverse events were recorded only when they led to discontinuation and therefore do not represent overall safety incidence. **Conclusions**: Golimumab was characterized by substantial 36-month treatment persistence and clinical improvement among patients who remained on treatment and were evaluable at week 144 (*n* = 88). Full-cohort NRI analyses also demonstrated clinically relevant responses at week 144. Comorbidity burden and poorer functional class were associated with discontinuation in exploratory analyses. These findings add long-term real-world evidence from an under-represented geographic setting.

## 1. Introduction

Axial spondyloarthritis (axSpA) is a chronic inflammatory rheumatic disease characterized by axial pain and stiffness, impaired function, and, in some patients, progressive structural damage. Contemporary management emphasizes regular assessment of disease activity and timely escalation to biologic or targeted synthetic disease-modifying antirheumatic drugs when active disease persists despite conventional treatment [1]. The epidemiology and clinical expression of spondyloarthritis vary across geographic regions, while several populations remain under-represented in international evidence bases [2,3].

Tumor necrosis factor inhibitors (TNFi) remain established treatment options for active axSpA. Golimumab, a fully human monoclonal antibody targeting tumor necrosis factor-α, has demonstrated efficacy in randomized trials, with sustained improvements in disease activity, physical function, and related outcomes during long-term extension follow-up [4,5,6,7]. These trials establish efficacy under protocolized conditions but do not fully describe how long patients continue therapy, why treatment is stopped, or which clinical characteristics are associated with persistence in routine practice.

Real-world treatment persistence is therefore a clinically useful complement to trial efficacy. It reflects the combined influence of effectiveness, tolerability, patient and physician decisions, treatment history, and the organization of care. In axSpA, persistence with TNFi varies substantially across cohorts and registries, and golimumab-specific data are less extensive than trial evidence [8,9,10,11]. Comorbidities and functional impairment may also affect treatment response and persistence independently of inflammatory activity [12,13].

Evidence from Central Asia is particularly limited. Kazakhstan has an expanding rheumatology infrastructure and government-funded biologic treatment programs, but long-term, locally generated data on TNFi persistence are sparse [14]. Country-specific evidence is important because retention estimates cannot be assumed to transfer directly across healthcare systems or patient populations. The present study therefore aimed to evaluate clinical outcomes during golimumab treatment through week 144 and treatment persistence through 36 months in patients with axSpA treated in routine clinical practice in Kazakhstan, to explore baseline factors associated with treatment discontinuation, and to describe reasons for discontinuation, including safety-related reasons.

## 2. Materials and Methods

### 2.1. Study Design and Setting

This was a prospective observational cohort study conducted at two rheumatology centers in Almaty, Kazakhstan: The Center of Internal Medicine of S.D. Asfendiyarov Kazakh National Medical University and the Almaty City Rheumatology Center. A total of 113 patients with axial spondyloarthritis (axSpA) were enrolled in October 2019 and prospectively followed in routine clinical practice through week 144 (2022). Standardized clinical assessments were performed at baseline (week 0) and at weeks 12, 24, 52, 104, and 144. Baseline (week 0) was the enrollment visit, at which all patients had already received golimumab for at least 12 weeks; the cohort therefore consisted of prevalent rather than incident (new) golimumab users. Time zero for all time-to-event analyses was the enrollment date, and the data cutoff for the prospective follow-up was December 2022. A separate descriptive treatment-status assessment was conducted in November 2025 (Section 2.6).

### 2.2. Participants and Treatment

**Figure 1:**
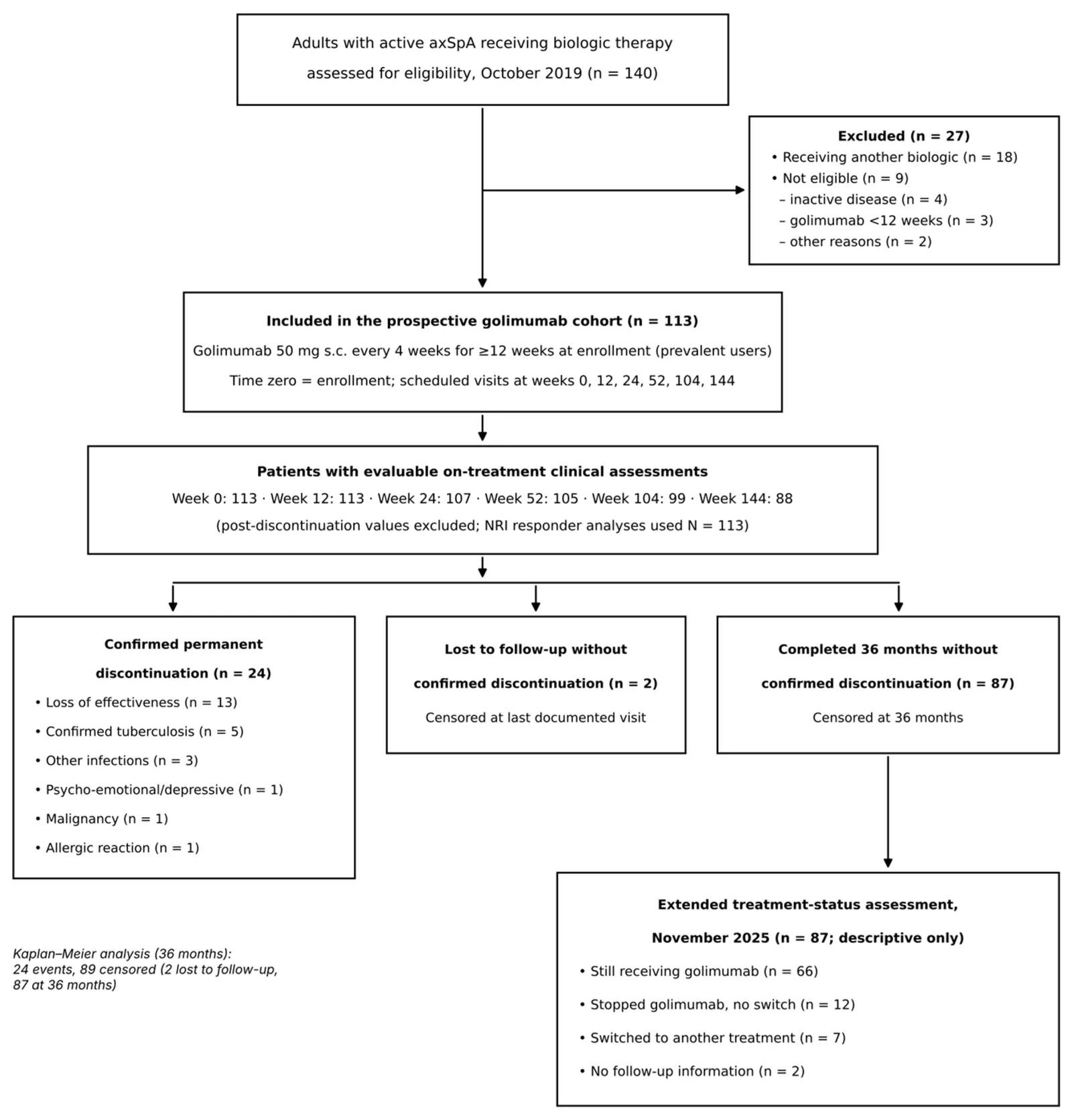
Flow diagram of patient screening, inclusion, follow-up, confirmed discontinuation, censoring, and the 2025 treatment-status assessment.

In October 2019, a total of 140 adults with active axSpA receiving biologic therapy at the two participating rheumatology centers were consecutively assessed for eligibility. Of these, 27 were excluded because they were receiving another biologic agent or did not meet the eligibility criteria for the golimumab cohort, leaving 113 patients for inclusion in the prospective cohort (Figure 1).

Eligible patients were aged ≥18 years, fulfilled the 2009 Assessment of SpondyloArthritis international Society (ASAS) classification criteria for axSpA [15], required biologic therapy as part of routine clinical care, and had been receiving golimumab 50 mg subcutaneously every four weeks for at least 12 weeks at the time of study enrollment.

Radiographic axSpA (r-axSpA) was defined according to the radiographic component of the modified New York (mNY) criteria as bilateral sacroiliitis grade II–IV or unilateral sacroiliitis grade III–IV. Patients with available radiographs who did not meet the mNY radiographic criterion were classified as having non-radiographic axSpA (nr-axSpA). In patients without available radiographs, radiographic status was considered undetermined and was not inferred from MRI findings alone. Based on the available imaging data, 54 patients fulfilled the mNY radiographic criterion, 19 had available radiographs but did not fulfill the mNY criterion, and radiographic status could not be determined in 40 patients. Among the latter, 29 had MRI data without radiography, whereas 11 had neither radiographic nor MRI data available.

Golimumab was administered at a dose of 50 mg subcutaneously every four weeks within the guaranteed volume of free medical care of the Republic of Kazakhstan.

### 2.3. Clinical Assessments

Clinical assessments were performed at baseline and weeks 12, 24, 52, 104, and 144. Standardized clinical outcomes were therefore assessed through week 144, whereas treatment persistence was evaluated separately through 36 months (approximately 156 weeks). These represent distinct follow-up time points. Disease activity was measured using the Bath Ankylosing Spondylitis Disease Activity Index (BASDAI) and the Ankylosing Spondylitis Disease Activity Score (ASDAS) based on erythrocyte sedimentation rate (ESR; ASDAS-ESR) [16,17]. ASDAS-ESR was used because ESR measurements were more consistently available at baseline and across scheduled follow-up visits than CRP measurements, allowing the same ASDAS formulation to be applied throughout the longitudinal analysis while minimizing missing data. Physical function was assessed using the Bath Ankylosing Spondylitis Functional Index (BASFI), spinal mobility using the Bath Ankylosing Spondylitis Metrology Index (BASMI), and enthesitis using the Maastricht Ankylosing Spondylitis Enthesitis Score (MASES) [18,19,20].

Baseline variables included age, sex, smoking status, disease duration, time since diagnosis, diagnostic delay, family history of spondyloarthritis, HLA-B27 status, functional class, previous exposure to conventional synthetic and biologic disease-modifying antirheumatic drugs (csDMARDs and bDMARDs), extra-musculoskeletal manifestations, and comorbidities. Laboratory assessments included erythrocyte sedimentation rate (ESR), C-reactive protein (CRP), and fecal calprotectin (µg/g), when available. Comorbidity burden was summarized using the Rheumatic Disease Comorbidity Index (RDCI) [21], calculated according to the standard validated scoring algorithm; osteoarthritis was not included in the RDCI calculation. Functional class was graded from I to IV according to the Clinical Protocol for the Diagnosis and Treatment of Ankylosing Spondylitis in force in the Republic of Kazakhstan at the time of cohort enrollment (Protocol No. 12, 29 September 2016 [22]). For the present analyses, functional class was dichotomized as III–IV versus I–II, with classes I–II as the reference category.

For patients receiving golimumab at a scheduled follow-up visit, clinical assessments were performed before the treating physician made a subsequent decision regarding treatment continuation or discontinuation. Assessments obtained immediately before a decision to discontinue golimumab were therefore retained as on-treatment observations. If golimumab had already been discontinued before a scheduled visit, no subsequent assessments were included in the on-treatment analyses.

Clinical outcome analyses at each follow-up visit included patients who remained on golimumab and had a valid clinical assessment at the corresponding time point. Values recorded after confirmed golimumab discontinuation or loss to follow-up were not included in the longitudinal clinical outcome analyses. The numbers of patients with valid on-treatment clinical assessments were 113 at baseline, 113 at week 12, 107 at week 24, 105 at week 52, 99 at week 104, and 88 at week 144. No imputation was performed for longitudinal continuous clinical outcomes.

### 2.4. Outcomes

The primary outcome was golimumab treatment persistence during the available 36-month observation period. Confirmed permanent discontinuation of golimumab for any documented reason was considered an event. Patients without confirmed treatment discontinuation were censored at their last documented follow-up. Patients who were lost to follow-up without confirmation of golimumab discontinuation were therefore treated as censored observations rather than as discontinuation events.

Secondary outcomes included longitudinal changes in disease activity (BASDAI and ASDAS-ESR), physical function (BASFI), spinal mobility (BASMI), and enthesitis (MASES) from baseline through week 144. Additional secondary outcomes included achievement of predefined clinical response states, documented reasons for golimumab discontinuation, and exploratory assessment of baseline factors associated with treatment discontinuation.

BASDAI50 response was defined as a ≥50% reduction in BASDAI from baseline. ASDAS inactive disease was defined as ASDAS-ESR < 1.3, and ASDAS major improvement as a decrease in ASDAS-ESR of ≥2.0 units from baseline.

### 2.5. Treatment Discontinuation and Safety-Related Events

Reasons for permanent golimumab discontinuation were classified as loss of effectiveness, tuberculosis, other infectious complications, psycho-emotional reasons/depression, malignancy, or allergic reaction. Loss of effectiveness was defined as an insufficient initial clinical response or a reduction in a previously achieved response that led the treating rheumatologist to discontinue golimumab or switch to another biologic therapy.

Before initiation of biologic therapy, patients underwent tuberculosis screening as part of routine clinical practice in accordance with the national standard for rheumatology care in the Republic of Kazakhstan. Pretreatment assessment included Diaskintest^®^ or QuantiFERON testing, chest radiography, and evaluation by a phthisiatrician to determine tuberculosis status and the absence of contraindications to biologic therapy. During treatment, patients underwent regular clinical, laboratory, and instrumental monitoring at 3–6-month intervals, including repeated tuberculosis screening. Patients with positive immunodiagnostic tests or suspicious radiographic findings were referred to a phthisiatrician for further diagnostic evaluation. A positive screening test or isolated radiographic abnormality alone was not considered diagnostic of tuberculosis. For the present study, tuberculosis was classified as confirmed only when the diagnosis had been established by a phthisiatrician and documented in the medical record. All five tuberculosis cases that resulted in permanent golimumab discontinuation were confirmed diagnoses.

Other infectious complications were defined as infections other than tuberculosis that led the treating rheumatologist to permanently discontinue golimumab and comprised two documented cases of COVID-19 and one case of hepatitis C. Malignancy was defined as a newly diagnosed malignancy during golimumab treatment; one patient was diagnosed with stage II right renal cancer (T2bNxM0). Allergic reaction was defined as a hypersensitivity reaction attributed to golimumab by the treating physician; one patient developed angioedema (Quincke’s edema). All of these events were documented in the medical records and resulted in permanent golimumab discontinuation.

Safety ascertainment in the present study was limited to adverse events documented as contributing to permanent golimumab discontinuation. The study did not systematically capture all adverse events occurring during golimumab exposure; therefore, safety-related events reported in this study should not be interpreted as estimates of the overall incidence of adverse events during golimumab treatment.

### 2.6. Extended Treatment-Status Assessment

In November 2025, an additional treatment-status assessment was conducted among the 87 patients who completed 36 months of follow-up without confirmed discontinuation. This assessment determined whether patients were still receiving golimumab, had discontinued golimumab without switching to another therapy, had switched to another treatment, or had no available follow-up information. Standardized clinical outcomes were not collected at this extended assessment. Because exact dates of treatment discontinuation or switching after the 36-month observation period were not consistently available, the 2025 data were analyzed descriptively and were not incorporated into the Kaplan–Meier or Cox regression analyses.

### 2.7. Statistical Analysis

Continuous variables were summarized as mean ± standard deviation (SD) or median with the interquartile range (IQR), as appropriate. Categorical variables were presented as frequencies and percentages. Available denominators were reported where data were missing. Missing baseline data were not imputed. For variables with missing observations, descriptive statistics and percentages were calculated using the number of patients with available data as the denominator, and the corresponding denominators are reported in Table 1 and Table 2. Therefore, denominators may vary across variables.

Longitudinal BASDAI, ASDAS-ESR, BASFI, BASMI, and MASES values were summarized descriptively at baseline and weeks 12, 24, 52, 104, and 144. Changes from baseline to week 144 were evaluated using paired Wilcoxon signed-rank tests among patients with valid paired observations. For these analyses, the number of paired observations and the median within-patient change with IQR were reported. No imputation was performed for longitudinal continuous outcomes.

For responder analyses, patients without an evaluable assessment at the corresponding follow-up visit, including those who had discontinued golimumab or were lost to follow-up before that visit, were classified as non-responders (non-responder imputation). Response proportions were therefore calculated using the original cohort of 113 patients as the denominator.

Golimumab treatment persistence was estimated using the Kaplan–Meier method and reported with 95% confidence intervals (CIs). Confirmed permanent discontinuation of golimumab was considered an event. Patients without confirmed discontinuation were censored at their last documented follow-up; this included patients lost to follow-up without confirmation of treatment discontinuation. Median follow-up was estimated using the reverse Kaplan–Meier method. Time was measured from the enrollment date. Because treatment status was ascertained at scheduled visits, the time of confirmed discontinuation was assigned to the visit at which it was documented; the Kaplan–Meier curve therefore changes in steps at visit time points. Time on golimumab before enrollment was not included in the treatment-persistence analysis. Accordingly, the estimates describe persistence after cohort enrollment among prevalent golimumab users rather than persistence from treatment initiation. Kaplan–Meier curves were compared between prespecified subgroups using the log-rank test.

Associations between baseline characteristics and confirmed golimumab discontinuation were examined using Cox proportional hazards regression. Univariable Cox models were first fitted for the candidate predictors. A limited multivariable model included the RDCI total score, functional class (III–IV vs. I–II), and previous csDMARD exposure before cohort entry, modeled as a three-level categorical variable (none, sulfasalazine, or methotrexate), with no previous csDMARD exposure as the reference category. This variable represented previous csDMARD treatment history and was distinct from the concomitant csDMARD regimen recorded at baseline. No patient had previous exposure to more than one csDMARD. The leflunomide use reported in Table 1 referred to concomitant treatment at baseline and was therefore not a category of the previous csDMARD exposure variable used in the Cox model. Candidate variables were selected on the basis of clinical relevance and their univariable association with discontinuation, and the number of covariates was deliberately restricted to limit overfitting; diagnostic delay and osteoarthritis were therefore not entered into the multivariable model. Because 24 confirmed discontinuation events occurred and four regression coefficients were estimated in the multivariable model, the analysis was considered exploratory. Results are reported as hazard ratios (HRs) with 95% CIs and *p*-values. The proportional hazards assumption was evaluated using Schoenfeld residuals and the global proportional hazards test.

A sensitivity analysis was performed using a restricted event definition that included discontinuations due to loss of effectiveness or adverse-event-related reasons. Discontinuation for psycho-emotional reasons/depression was censored at the corresponding observed treatment time.

All statistical tests were two-sided, and *p* < 0.05 was considered a nominal threshold for statistical significance. No adjustment for multiple comparisons was performed. Statistical analyses were performed using R version 4.3.2 (R Foundation for Statistical Computing, Vienna, Austria), including the survival and survminer packages. The study was reported in accordance with the STROBE guidelines for cohort studies (Supplementary Table S1).

### 2.8. Ethics Statement

The study was conducted in accordance with the Declaration of Helsinki and Good Clinical Practice. The study protocol was approved by the Local Ethics Committee of S.D. Asfendiyarov Kazakh National Medical University (Protocol No. 8 [87], 25 September 2019). All participants provided written informed consent for participation in the study and for the processing of personal data.

## 3. Results

### 3.1. Study Population and Baseline Characteristics

A total of 140 adults with active axSpA receiving biologic therapy were assessed for eligibility in October 2019. Of these, 27 were excluded because they were receiving another biologic agent or did not meet the eligibility criteria for the golimumab cohort, leaving 113 patients for inclusion in the prospective cohort (Figure 1).

During the 36-month treatment-persistence observation period, 24 patients had a confirmed permanent discontinuation of golimumab, whereas 89 had no confirmed discontinuation; the latter group included two patients who were lost to follow-up and were censored at their last documented follow-up. Thus, 87 patients completed the 36-month observation period without confirmed discontinuation (Figure 1).

Patients with confirmed discontinuation had a higher baseline RDCI score than those without confirmed discontinuation (median 3.0 [IQR 1.0–3.2] vs. 1.0 [0.0–3.0]; *p* = 0.003), more frequently had functional class III–IV (37.5% vs. 11.2%; *p* = 0.005), and had a longer diagnostic delay (median 8.5 [3.0–12.0] vs. 5.0 [1.0–9.0] years; *p* = 0.043). Other demographic, disease-history, and treatment characteristics were broadly similar between groups (Table 1). Golimumab was used as the first biologic in 86/113 (76.1%) patients and as a later-line biologic in 27/113 (23.9%). At baseline, 84/113 (74.3%) patients were receiving a concomitant csDMARD. Regarding radiographic classification, 54/113 (47.8%) patients fulfilled the modified New York radiographic criterion and were classified as r-axSpA, 19/113 (16.8%) had available radiographs that did not fulfill the criterion and were classified as nr-axSpA, while radiographic status remained undetermined in 40/113 (35.4%) because appropriate radiographs were unavailable.

Baseline disease activity, functional measures, and inflammatory markers did not differ significantly between groups. Osteoarthritis was more frequent among patients with confirmed discontinuation (47.6% vs. 20.2%; *p* = 0.023), whereas the other evaluated comorbidities and extra-musculoskeletal manifestations did not differ significantly (Table 2). These baseline comparisons were exploratory and unadjusted and should not be interpreted as independent predictors of treatment discontinuation.

### 3.2. Golimumab Treatment Persistence

During the 36-month treatment-persistence observation period, 24 confirmed permanent discontinuations of golimumab were recorded. Two patients were lost to follow-up without confirmed treatment discontinuation and were therefore censored at their last documented follow-up.

**Figure 2:**
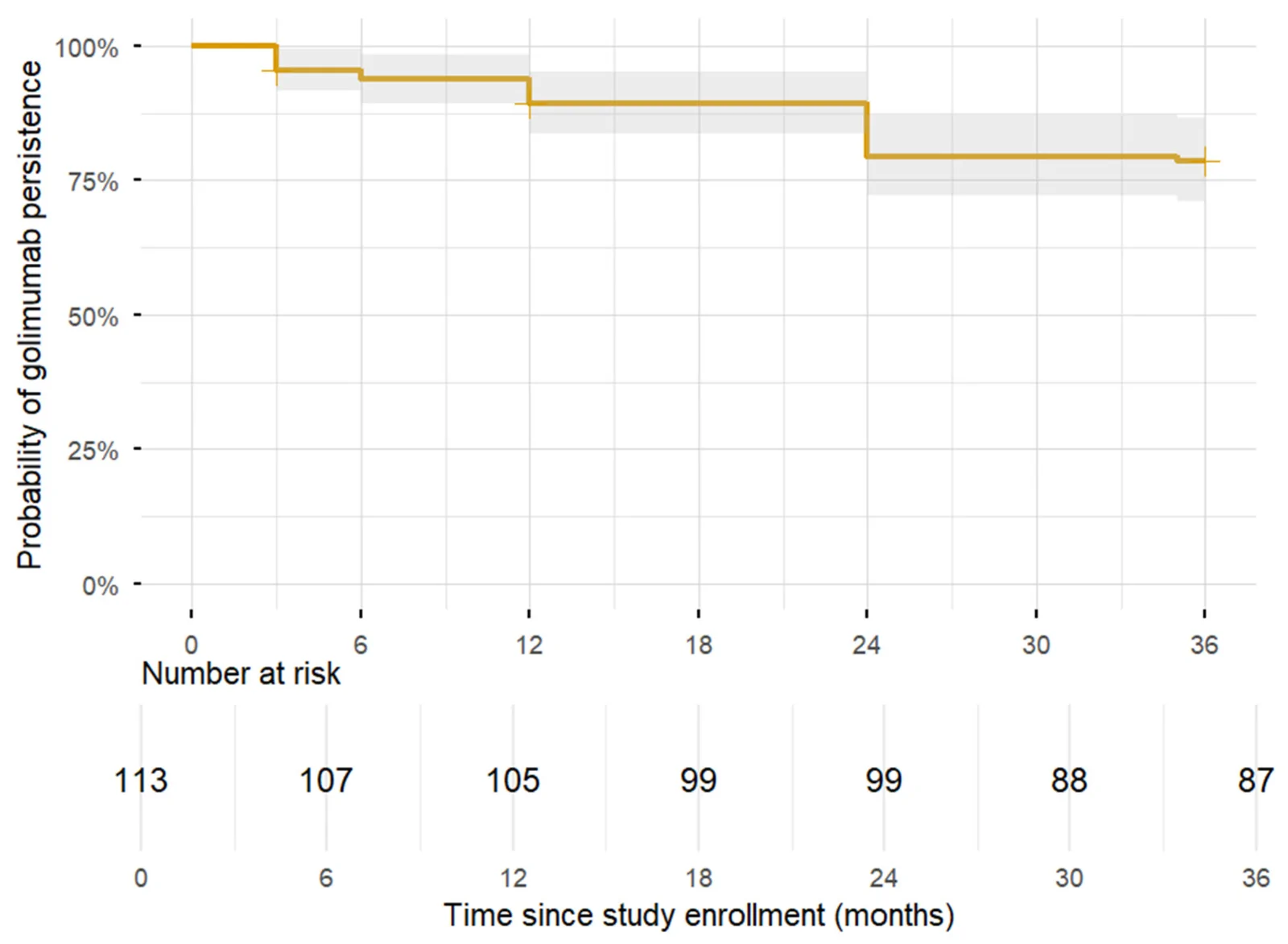
Kaplan–Meier estimate of golimumab treatment persistence over 36 months. The shaded area represents the 95% confidence interval, and tick marks indicate censored observations. Patients lost to follow-up without confirmed treatment discontinuation were censored at their last documented follow-up. Numbers at risk are shown below the plot and represent patients at risk immediately before discontinuation events and censoring at each indicated time point, whereas the Kaplan–Meier estimates reflect treatment persistence after accounting for these events. Time is measured from study enrollment; discontinuations were dated to the scheduled visit at which they were documented.

Kaplan–Meier estimated treatment persistence was 93.8% (95% CI, 89.4–98.4%) at 6 months, 89.3% (95% CI, 83.8–95.2%) at 12 months, and 79.4% (95% CI, 72.2–87.3%) at 24 months. At week 144 (approximately 33.2 months), estimated persistence remained 79.4% (95% CI, 72.2–87.3%). At 36 months, the Kaplan–Meier estimate was 78.5% (95% CI, 71.2–86.5%), and 87 patients completed 36 months without confirmed discontinuation (Figure 2).

The median treatment-persistence time was not reached during follow-up. Median follow-up, estimated using the reverse Kaplan–Meier method, was 36 months.

### 3.3. Factors Associated with Treatment Discontinuation

In univariable Cox proportional hazards analyses, a higher RDCI score was associated with a higher hazard of confirmed golimumab discontinuation (HR 1.357, 95% CI 1.085–1.696; *p* = 0.007), as was functional class III–IV compared with functional class I–II (HR 3.641, 95% CI 1.590–8.334; *p* = 0.002). Previous csDMARD history was also associated with discontinuation in the overall likelihood-ratio test (*p* = 0.024); however, the individual comparisons of previous sulfasalazine and methotrexate use with no previous csDMARD use did not reach statistical significance (Table 3).

**Figure 3:**
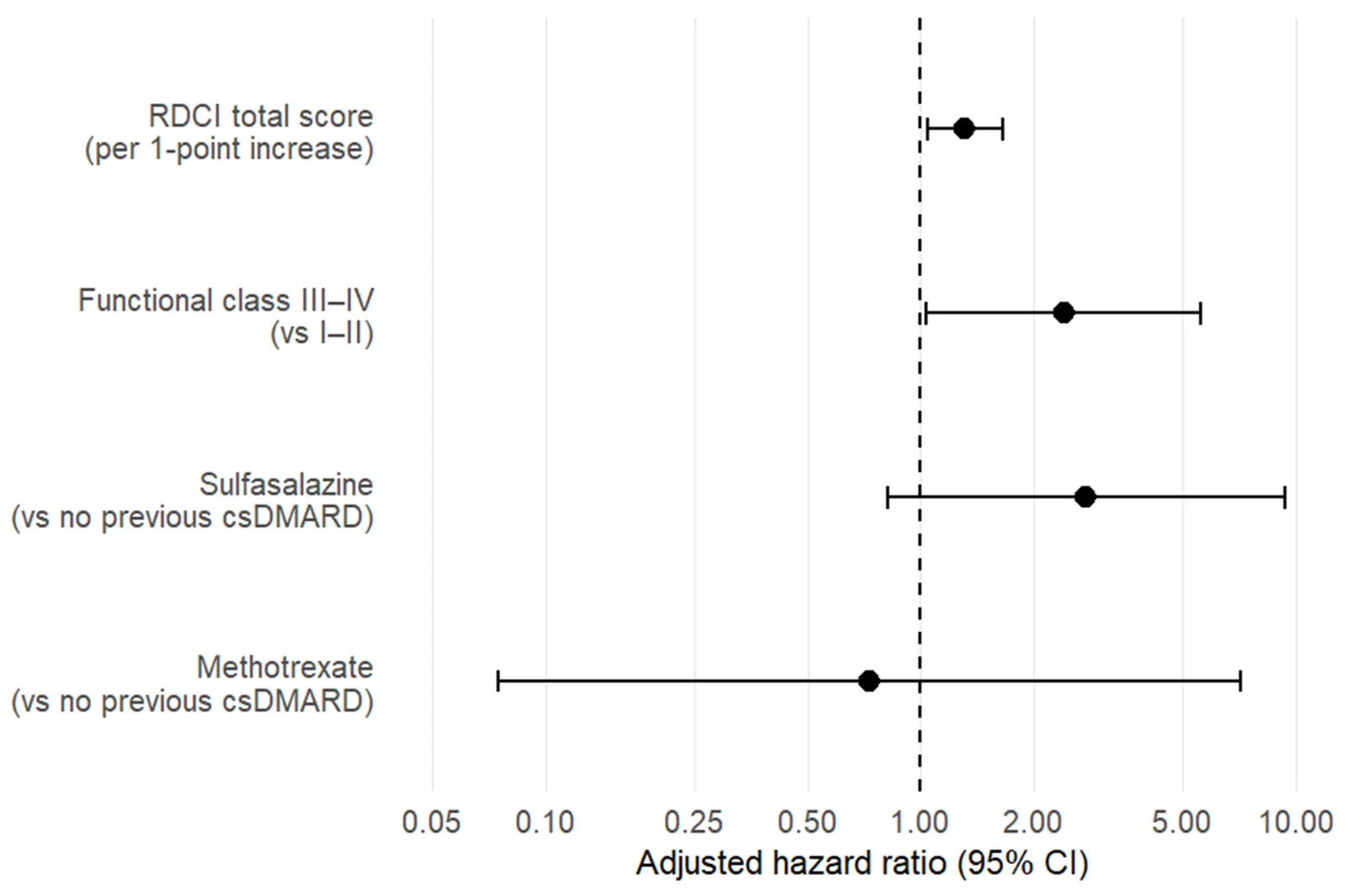
Adjusted hazard ratios for confirmed golimumab discontinuation from the exploratory multivariable Cox proportional hazards model. Points represent adjusted hazard ratios and horizontal bars indicate 95% confidence intervals. The vertical dashed line indicates a hazard ratio of 1.0. No previous csDMARD use was the reference category for previous csDMARD history. RDCI, Rheumatic Disease Comorbidity Index; csDMARD, conventional synthetic disease-modifying antirheumatic drug.

In the exploratory multivariable Cox model, each one-point increase in RDCI was associated with a higher hazard of confirmed golimumab discontinuation (adjusted HR 1.312, 95% CI 1.042–1.653; *p* = 0.021). Functional class III–IV was also associated with a higher hazard of discontinuation compared with functional class I–II (adjusted HR 2.412, 95% CI 1.037–5.611; *p* = 0.041). Previous csDMARD history was not independently associated with discontinuation in the overall likelihood-ratio test (*p* = 0.086). Adjusted hazard ratios from the multivariable model are shown in Figure 3.

The proportional hazards assumption showed no evidence of violation for RDCI (*p* = 0.94), functional class (*p* = 0.76), previous csDMARD history (*p* = 0.50), or the model globally (*p* = 0.82). Given the limited number of confirmed discontinuation events (*n* = 24) relative to the four model coefficients, the multivariable analysis should be interpreted as exploratory.

### 3.4. Clinical Outcomes Through Week 144

**Figure 4:**
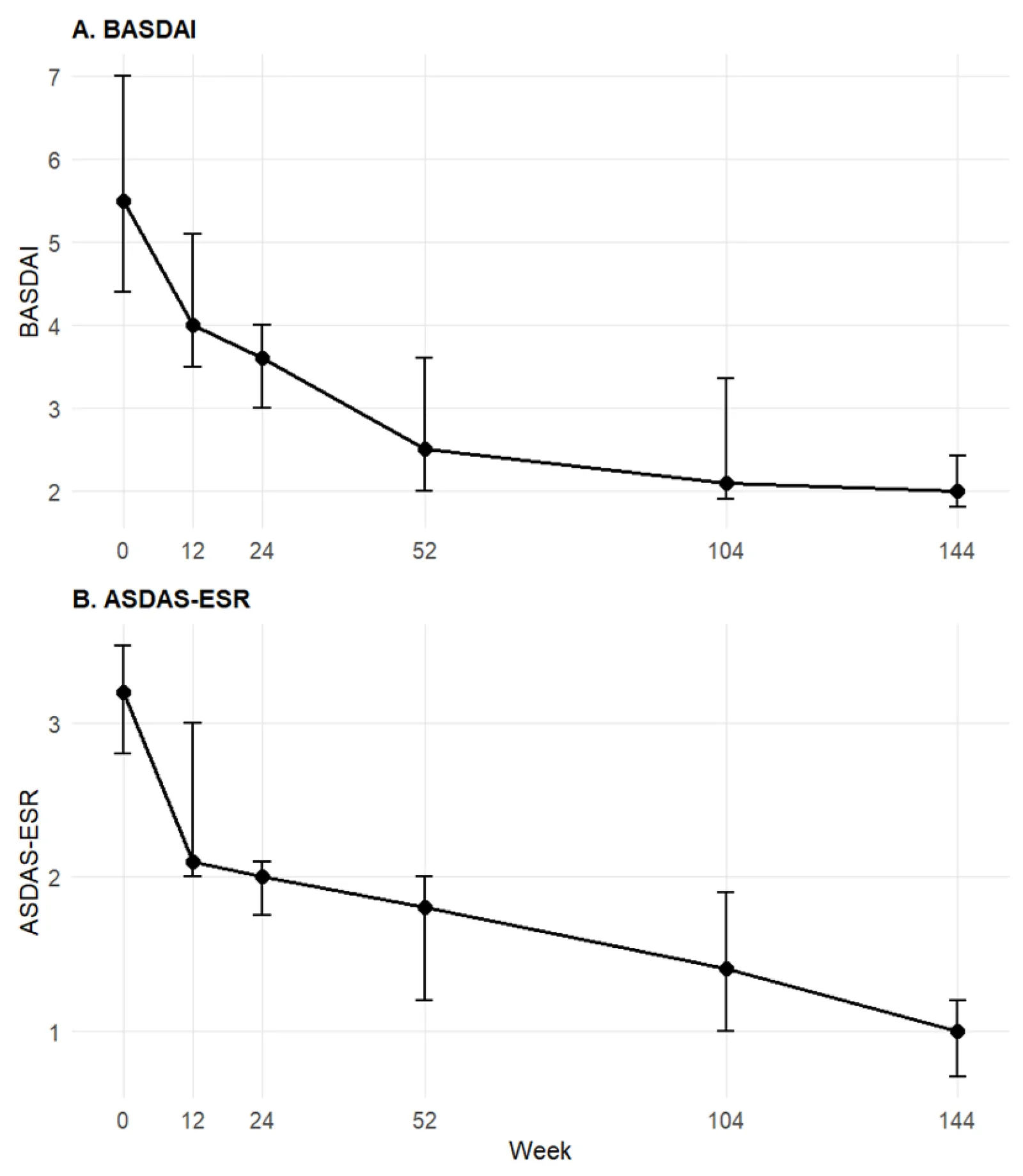
Changes in disease activity during prospective follow-up. (**A**) BASDAI and (**B**) ASDAS-ESR scores from baseline through week 144. Points represent medians and error bars represent interquartile ranges among patients with available on-treatment assessments at each visit. The numbers of evaluable patients were 113 at baseline and week 12, 107 at week 24, 105 at week 52, 99 at week 104, and 88 at week 144. Post-discontinuation measurements were excluded.

**Figure 5:**
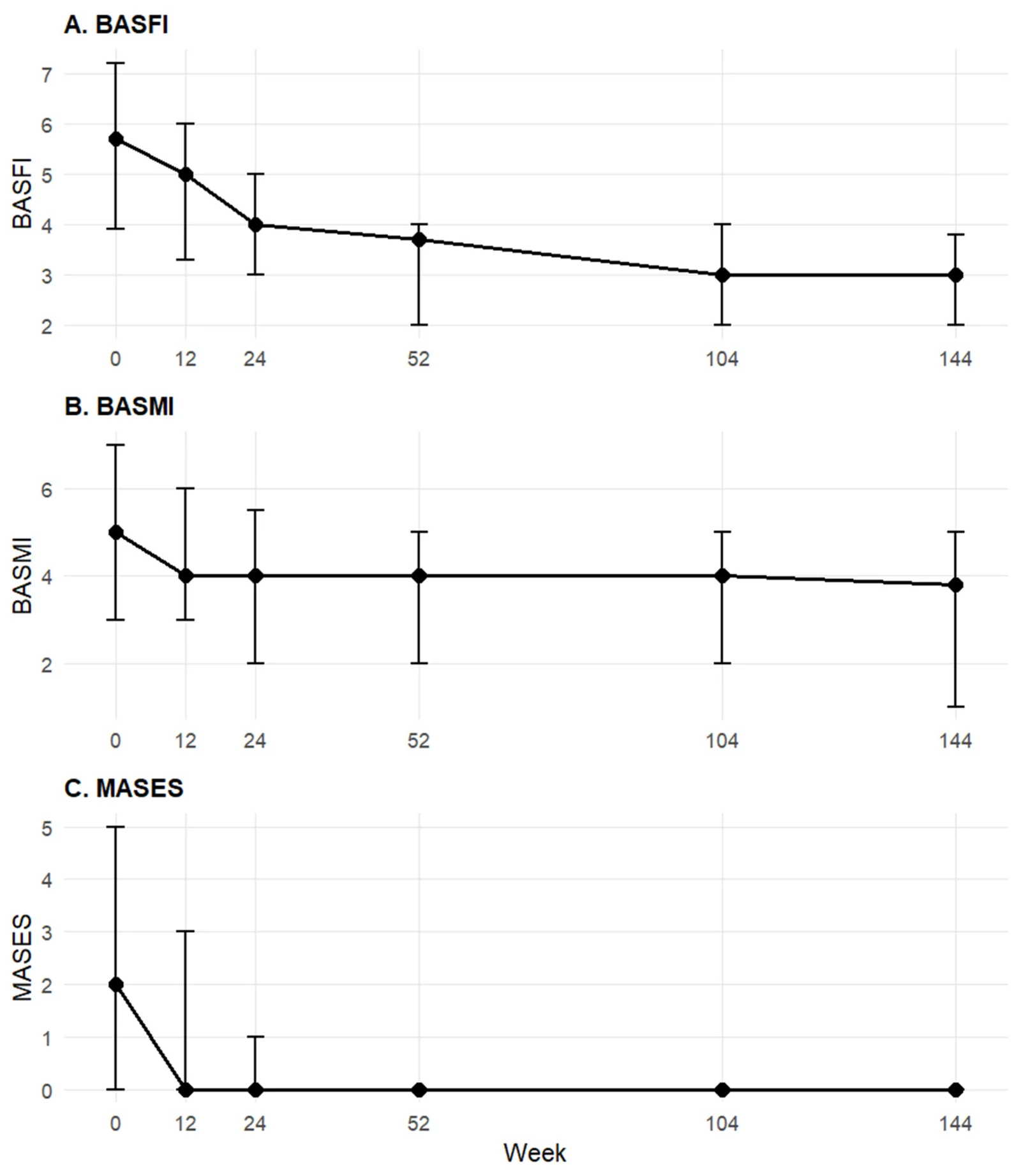
Changes in functional status, spinal mobility, and enthesitis during prospective follow-up. (**A**) BASFI, (**B**) BASMI, and (**C**) MASESs from baseline through week 144. Points represent medians and error bars represent interquartile ranges among patients with available on-treatment assessments at each visit. The numbers of evaluable patients were 113 at baseline and week 12, 107 at week 24, 105 at week 52, 99 at week 104, and 88 at week 144. Post-discontinuation measurements were excluded.

Clinical outcomes improved over the prospective follow-up among patients who remained on golimumab and had valid on-treatment assessments. Evaluable clinical data were available for 113 patients at baseline and week 12, 107 at week 24, 105 at week 52, 99 at week 104, and 88 at week 144 (Table 4; Figure 4 and Figure 5).

Among the 88 patients with paired baseline and week 144 assessments, median BASDAI decreased from 5.5 to 2.0, with a median paired change of −3.35 (IQR −5.00 to −2.38; *p* < 0.001). Median ASDAS-ESR decreased from 3.3 to 1.0, corresponding to a median paired change of −2.20 (IQR −2.50 to −1.90; *p* < 0.001) (Figure 4).

Functional and clinical measures also changed over follow-up. Median BASFI decreased from 5.8 to 3.0 among patients with paired assessments (median change −2.65, IQR −4.13 to −1.20; *p* < 0.001), while median BASMI decreased from 5.0 to 3.8 (median change −1.00, IQR −2.00 to −0.08; *p* < 0.001). Median MASES decreased from 2.0 at baseline to 0 at week 144 (median change −2.00, IQR −5.00 to 0.00; *p* < 0.001) (Figure 5).

These longitudinal analyses represent observed on-treatment outcomes. Patients who permanently discontinued golimumab did not contribute clinical measurements after discontinuation; therefore, later follow-up estimates reflect the subset of patients remaining under observation and on treatment and may be affected by attrition and survivor bias.

### 3.5. Clinical Response Outcomes

Clinical response rates increased over the follow-up period using non-responder imputation (NRI), with all patients in the baseline cohort (N = 113) retained as the denominator at each time point. Patients without an available on-treatment assessment at a given visit were classified as non-responders.

**Figure 6:**
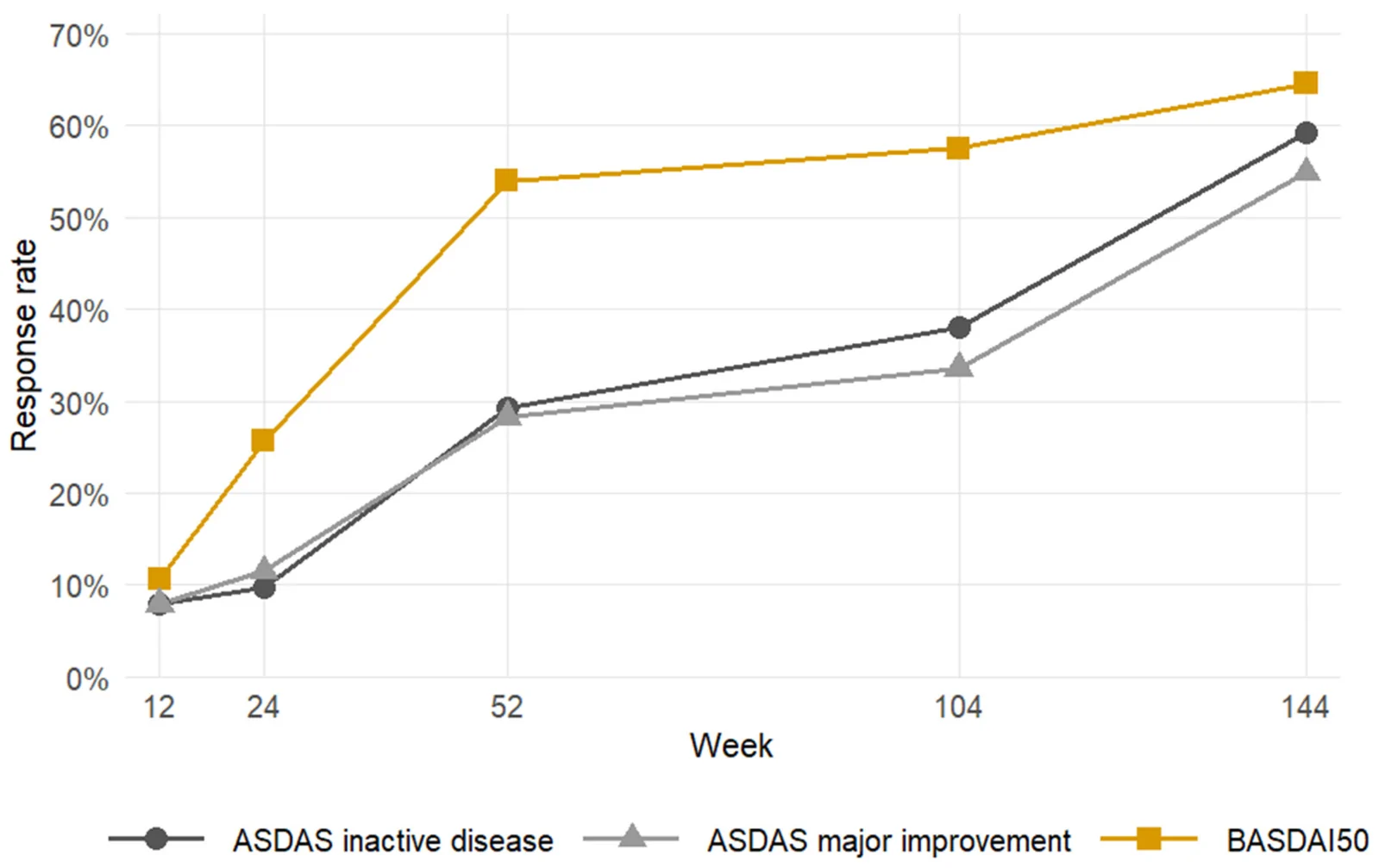
Clinical response outcomes through week 144 using non-responder imputation. Response rates for BASDAI50, ASDAS inactive disease, and ASDAS major improvement are shown at weeks 12, 24, 52, 104, and 144. All response rates were calculated using the baseline cohort (N = 113) as the denominator; patients without an available on-treatment assessment at a given visit were classified as non-responders. BASDAI50 was defined as a ≥50% reduction from baseline in BASDAI; ASDAS inactive disease as ASDAS-ESR < 1.3; and ASDAS major improvement as a decrease in ASDAS-ESR of ≥2.0 from baseline.

The proportion of patients achieving BASDAI50 increased from 10.6% (12/113) at week 12 to 25.7% (29/113) at week 24 and 54.0% (61/113) at week 52, reaching 57.5% (65/113) at week 104 and 64.6% (73/113) at week 144 (Figure 6).

ASDAS inactive disease was achieved by 8.0% (9/113) of patients at week 12, 9.7% (11/113) at week 24, 29.2% (33/113) at week 52, 38.1% (43/113) at week 104, and 59.3% (67/113) at week 144. ASDAS major improvement was observed in 8.0% (9/113), 11.5% (13/113), 28.3% (32/113), 33.6% (38/113), and 54.9% (62/113) of patients at weeks 12, 24, 52, 104, and 144, respectively (Figure 6).

### 3.6. Reasons for Treatment Discontinuation and Sensitivity Analysis

Among the 24 patients with confirmed permanent golimumab discontinuation, the most frequent reason was loss of effectiveness, reported in 13 patients (54.2%). Treatment was discontinued because of confirmed tuberculosis in 5 patients (20.8%) and other infectious complications in 3 patients (12.5%), comprising two cases of COVID-19 and one case of hepatitis C. The remaining discontinuations were attributed to psycho-emotional/depressive symptoms in 1 patient (4.2%), malignancy (stage II right renal cancer, T2bNxM0) in 1 patient (4.2%), and an allergic reaction (angioedema) in 1 patient (4.2%) (Table 5).

A sensitivity analysis was performed using a more restrictive discontinuation endpoint that included discontinuations due to loss of effectiveness or adverse-event-related reasons (confirmed tuberculosis, other infectious complications, malignancy, or allergic reaction), while the discontinuation attributed to psycho-emotional/depressive symptoms was censored. This analysis included 23 events. Kaplan–Meier estimated treatment persistence was 93.8% (95% CI, 89.4–98.4%) at 6 months, 89.3% (95% CI, 83.8–95.2%) at 12 months, 80.3% (95% CI, 73.2–88.0%) at 24 months, and 79.4% (95% CI, 72.2–87.3%) at 36 months. These estimates were similar to those obtained in the primary analysis, in which 24 confirmed discontinuations were treated as events.

### 3.7. Extended Treatment Status in 2025

Among the same 87 patients who completed 36 months without confirmed discontinuation, treatment status was additionally assessed in 2025. Of these, 66 (75.9%) were still receiving golimumab, 12 (13.8%) were no longer receiving golimumab without a documented switch to another therapy, and 7 (8.0%) had switched from golimumab to another treatment. Follow-up information was unavailable for 2 patients (2.3%). Because exact dates of treatment discontinuation or switching after 36 months were not consistently available, these findings represent a descriptive extended treatment-status assessment rather than Kaplan–Meier estimates of long-term persistence.

## 4. Discussion

This prospective real-world cohort provides long-term data on golimumab treatment persistence and clinical outcomes in patients with axial spondyloarthritis (axSpA) treated in routine clinical practice in Kazakhstan. Golimumab persistence was 78.5% (95% CI, 71.2–86.5%) at 36 months. Among patients with evaluable assessments, improvements in disease activity, physical function, spinal mobility, and enthesitis were observed through week 144; complementary non-responder imputation analyses using the full baseline cohort also demonstrated clinically relevant response rates. Higher comorbidity burden and functional class III–IV were associated with a higher hazard of confirmed discontinuation in exploratory analyses.

The 36-month persistence estimate of 78.5% observed in our cohort indicates that a substantial proportion of patients continued golimumab during long-term routine care. An additional descriptive treatment-status assessment in 2025 showed that, among the 87 patients who completed 36 months without confirmed discontinuation, 66 (75.9%) were still receiving golimumab, 12 (13.8%) were no longer receiving golimumab, 7 (8.0%) had switched to another treatment, and follow-up information was unavailable for 2 (2.3%). Because exact dates of discontinuation or switching after 36 months were not consistently available, these data were not incorporated into the Kaplan–Meier analysis and should be interpreted as a descriptive extended treatment-status assessment rather than as a formal persistence estimate. Direct comparison with other real-world studies requires caution because study populations, treatment lines, definitions of persistence, and starting points of follow-up differ. In the prospective GO-PRACTICE study, the Kaplan–Meier estimate of golimumab persistence in patients with axSpA was 52.6% at 24 months [8], and 59.2% among biologic-naïve patients [8], which is the more relevant comparison for the present cohort, in which golimumab was the first biologic in 76.1% of patients. Importantly, GO-PRACTICE evaluated persistence from the initial golimumab prescription, whereas all patients in the present cohort had already received golimumab for at least 12 weeks at study enrollment. Our cohort therefore represents patients who had successfully remained on treatment through this initial exposure period, which may partly contribute to the higher observed persistence. Previous biologic exposure was also associated with lower persistence in GO-PRACTICE [8], while other European observational and registry studies have demonstrated substantial variability in TNFi and golimumab retention according to treatment line, patient characteristics, and clinical setting [9,10,11]. Accordingly, the present estimate should be interpreted as a description of persistence within this specific cohort and healthcare context rather than as evidence of superior golimumab persistence compared with other settings.

The longitudinal clinical findings were consistent with sustained improvement among patients remaining on golimumab. Among the 88 patients with paired baseline and week 144 assessments, median BASDAI decreased from 5.5 to 2.0 and median ASDAS-ESR from 3.3 to 1.0, with corresponding improvements in physical function, spinal mobility, and enthesitis. The direction of these changes is consistent with the sustained clinical benefit reported in long-term golimumab studies, including the 104-week and 5-year GO-RAISE follow-up and observational registry data [5,6,23]. However, the magnitude of improvement observed at later visits in the present study requires caution. Continuous longitudinal analyses were based on available on-treatment assessments, and patients who discontinued golimumab did not contribute subsequent measurements. Later visits may therefore be enriched for patients with a favorable treatment course, resulting in attrition and survivor bias. The median values at week 144 (BASDAI 2.0 and ASDAS-ESR 1.0) are lower than those generally reported in long-term trial extensions and registries and therefore describe a selected group of patients who remained on golimumab and under follow-up, not the full cohort. In addition, because enrollment required active disease after at least 12 weeks of golimumab, part of the subsequent improvement may reflect regression to the mean and should not be attributed to golimumab alone.

The responder analyses provide a complementary and more conservative perspective because all 113 patients enrolled at baseline remained in the denominator at each follow-up visit. Using non-responder imputation, BASDAI50 was achieved by 64.6% of the cohort at week 144, while 59.3% achieved ASDAS inactive disease and 54.9% achieved ASDAS major improvement. Patients without an evaluable on-treatment assessment at a given visit, including those who had discontinued treatment or were lost to follow-up, were classified as non-responders. These response estimates should therefore be considered alongside, rather than interchangeably with, the available-case trajectories of continuous disease-activity measures. Reporting both approaches provides a more transparent representation of long-term outcomes in the presence of treatment discontinuation and incomplete follow-up.

Comorbidity burden emerged as an important correlate of treatment discontinuation. Each one-point increase in RDCI was associated with a 31% higher hazard of confirmed golimumab discontinuation in the exploratory multivariable model (adjusted HR 1.312, 95% CI 1.042–1.653). Comorbidities are common in axSpA and have been associated with worse disease activity, physical function, quality of life, and other adverse health outcomes [12]. Previous registry evidence has also demonstrated associations between individual comorbidities and TNFi drug retention [13]. The present findings extend these observations by suggesting that overall comorbidity burden, summarized using the RDCI, may be relevant to long-term golimumab persistence. Nevertheless, the limited number of discontinuation events and the exploratory nature of the model preclude interpretation of RDCI as a validated prognostic marker on the basis of this cohort alone.

Functional class III–IV was also associated with a higher hazard of confirmed discontinuation compared with functional class I–II (adjusted HR 2.412, 95% CI 1.037–5.611). Functional limitation in axSpA can reflect several overlapping components, including inflammatory disease activity, accumulated structural impairment, deconditioning, degenerative musculoskeletal disease, and comorbidity. Of note, osteoarthritis was more frequent at baseline among patients who subsequently had confirmed discontinuation; however, this was an exploratory unadjusted comparison and should not be interpreted as an independent predictor. Together, these observations suggest that persistent symptoms and functional impairment during biologic treatment may not necessarily reflect inflammatory activity alone and support comprehensive assessment of comorbid and non-inflammatory contributors when treatment response is considered inadequate [1,12].

Loss of effectiveness was the most frequent reason for permanent golimumab discontinuation, accounting for 13 of 24 confirmed discontinuations (54.2%). This is broadly consistent with real-world evidence showing primary or secondary non-response as major reasons for golimumab discontinuation [8]. Safety-related reasons in the present cohort included five cases of confirmed tuberculosis, three other infectious complications, one malignancy, and one allergic reaction. These findings should not be interpreted as estimates of adverse-event incidence because the study captured safety events only when they contributed to permanent treatment discontinuation. Nevertheless, the occurrence of confirmed tuberculosis among discontinuations is clinically relevant and reinforces the importance of established tuberculosis screening and monitoring procedures in patients receiving biologic therapy. Current EULAR recommendations support screening for latent tuberculosis before initiation of biologic DMARDs and recommend consideration of national or regional epidemiological circumstances [24]. The present findings do not establish a new golimumab safety signal but underscore the continued relevance of infection-risk assessment during long-term biologic treatment.

The sensitivity analysis supported the robustness of the primary persistence estimate to the definition of discontinuation. When the endpoint was restricted to discontinuations due to loss of effectiveness or adverse-event-related reasons and the discontinuation attributed to psycho-emotional/depressive symptoms was censored, 23 events remained and estimated persistence at 36 months was 79.4% (95% CI, 72.2–87.3%), compared with 78.5% (95% CI, 71.2–86.5%) in the primary analysis. The small difference between these estimates indicates that the overall persistence findings were not materially affected by inclusion of the single discontinuation attributed to psycho-emotional/depressive symptoms.

The present study adds longitudinal real-world evidence from Kazakhstan, a country that remains under-represented in international axSpA cohorts [2,3]. In addition to 36-month treatment-persistence data, the cohort provides repeated standardized assessments of disease activity, physical function, spinal mobility, and enthesitis through week 144, thereby adding data from a comparatively under-represented geographic setting to the evidence base for long-term TNFi treatment. Published real-world evidence on long-term golimumab treatment in patients with axSpA from Central Asia remains limited. The study also evaluates the association of overall comorbidity burden, measured using the RDCI, with golimumab discontinuation and documents confirmed tuberculosis among the reasons for treatment discontinuation. Golimumab was provided within Kazakhstan’s publicly funded healthcare system; however, healthcare-system and access-related factors were not directly evaluated in this study and therefore cannot be considered explanatory factors for the observed treatment persistence.

### 4.1. Clinical Implications

The findings support assessment of long-term biologic treatment using both disease-specific measures and broader patient characteristics. Disease activity remains central to treatment monitoring, but comorbidity burden and functional limitation may provide additional information when evaluating patients at risk of treatment discontinuation. In patients with persistent symptoms despite biologic therapy, assessment of comorbidities and non-inflammatory contributors to pain and disability may help distinguish inadequate inflammatory control from other causes of impaired function [1,12]. At the same time, the associations observed in this cohort were exploratory and should not be used as thresholds for individual treatment decisions without external validation.

### 4.2. Strengths and Limitations

The main strengths of this study include its prospective observational design, repeated standardized clinical assessments through week 144, treatment-persistence follow-up through 36 months, explicit censoring of patients lost to follow-up without confirmed discontinuation, and use of both available-case continuous outcomes and non-responder imputation for categorical clinical responses. The study also provides real-world evidence from Kazakhstan, a setting that remains under-represented in international axSpA research [2,3].

Several limitations should be considered. First, the observational design and absence of an active comparator preclude causal or comparative-effectiveness conclusions. Second, recruitment from two centers in Almaty limits generalizability to the wider population of patients with axSpA in Kazakhstan. Third, only 24 confirmed discontinuation events occurred, limiting the complexity and precision of the multivariable Cox model; the identified associations should therefore be considered exploratory. Fourth, longitudinal continuous outcomes were based on available on-treatment assessments and are susceptible to attrition and survivor bias, although responder analyses using non-responder imputation were additionally performed to provide a more conservative assessment. Fifth, all participants had already received golimumab for at least 12 weeks before enrollment, introducing potential prevalent-user selection bias because patients who discontinued treatment earlier were not represented in the cohort. Consequently, the treatment-persistence estimates reflect persistence after cohort enrollment rather than from treatment initiation, limiting direct comparisons with inception cohorts. Sixth, the 2025 extended treatment-status assessment was limited to ascertainment of current treatment status; standardized clinical outcomes were not collected, and exact dates of discontinuation or switching after 36 months were not consistently available. Therefore, these data were analyzed descriptively and cannot be interpreted as a formal long-term persistence estimate or as evidence of sustained clinical effectiveness. Seventh, radiographic status could not be determined in 40 patients because appropriate radiographs were unavailable, limiting analyses according to r-axSpA versus nr-axSpA. Eighth, because enrollment required active disease after at least 12 weeks of golimumab, part of the subsequent improvement may reflect regression to the mean. Ninth, treatment status was ascertained at scheduled visits, so discontinuation times were approximated by visit dates. Finally, safety ascertainment was restricted to adverse events contributing to permanent discontinuation; therefore, the study cannot estimate the overall incidence of adverse events or provide comparative safety estimates.

## 5. Conclusions

In this prospective two-center observational cohort from Kazakhstan, golimumab was characterized by substantial treatment persistence through 36 months, with a Kaplan–Meier estimate of 78.5%. Among patients with evaluable on-treatment follow-up, improvements in disease activity, physical function, spinal mobility, and enthesitis were observed through week 144. Using non-responder imputation in the full baseline cohort, 64.6% achieved BASDAI50, 59.3% achieved ASDAS inactive disease, and 54.9% achieved ASDAS major improvement at week 144. In the descriptive treatment-status assessment conducted in 2025, 66 of the 87 patients who completed 36 months without confirmed discontinuation were confirmed to still be receiving golimumab. Higher comorbidity burden and functional class III–IV were associated with a higher hazard of confirmed treatment discontinuation in exploratory analyses. Because adverse events were recorded only when they led to discontinuation, these data do not estimate the overall safety of golimumab. These findings provide long-term real-world evidence from an under-represented geographic setting; however, the extended 2025 assessment should be interpreted descriptively and not as a formal persistence estimate or evidence of sustained clinical effectiveness.

## Figures and Tables

**Table 1:** Baseline demographic, disease, and treatment characteristics according to golimumab discontinuation status.

Characteristic	No Confirmed Discontinuation (N = 89)	Confirmed Discontinuation (N = 24)	*p*-Value
Demographic characteristics			
Age, years	38.0 (30.0–48.0)	41.0 (33.8–47.5)	0.347
Male sex, *n*/N (%)	66/89 (74.2)	20/24 (83.3)	0.428
Current smoking, *n*/N (%)	48/89 (53.9)	18/24 (75.0)	0.101
Disease history			
Disease duration, years	12.0 (7.0–19.0)	13.0 (9.0–19.5)	0.323
Time since diagnosis, years	5.0 (2.0–10.0)	4.0 (1.0–9.2)	0.789
Diagnostic delay, years	5.0 (1.0–9.0)	8.5 (3.0–12.0)	0.043
Family history of spondyloarthritis, *n*/N (%)	19/88 (21.6)	2/24 (8.3)	0.236
History of trauma, *n*/N (%)	26/89 (29.2)	6/24 (25.0)	0.802
Clinical characteristics			
Functional class III–IV, *n*/N (%)	10/89 (11.2)	9/24 (37.5)	0.005
RDCI total score	1.0 (0.0–3.0)	3.0 (1.0–3.2)	0.003
HLA-B27 positive, n/N (%)	72/78 (92.3)	19/20 (95.0)	1.000
Treatment characteristics			
Previous csDMARD use, *n*/N (%)	64/89 (71.9)	21/24 (87.5)	0.182
Concomitant csDMARD regimen at baseline, *n*/N (%)			0.802
None	23 (25.8)	6 (25.0)	
Sulfasalazine	55 (61.8)	14 (58.3)	
Methotrexate	10 (11.2)	4 (16.7)	
Leflunomide	1 (1.1)	0 (0.0)	
Concomitant csDMARD use, *n*/N (%)	66/89 (74.2)	18/24 (75.0)	1.000
Golimumab as later-line biologic, n (%)	19 (21.3)	8 (33.3)	0.281

Note. Values are median (25th–75th percentile) unless otherwise indicated. Percentages are based on available observations; denominators are shown where data were missing. *p*-values represent exploratory, unadjusted between-group comparisons. Continuous variables were compared using the Wilcoxon rank-sum test and categorical variables using Fisher’s exact test. bDMARD, biologic disease-modifying antirheumatic drug; csDMARD, conventional synthetic disease-modifying antirheumatic drug; HLA-B27, human leukocyte antigen B27; RDCI, Rheumatic Disease Comorbidity Index. Data are presented using available-case denominators. Denominators may vary between variables because of missing baseline data; no imputation was performed for missing baseline values.

**Table 2:** Baseline clinical and laboratory characteristics by golimumab retention status.

Characteristic	No Confirmed Discontinuation (N = 89)	Confirmed Discontinuation (N = 24)	*p*-Value
Disease activity and function			
BASDAI	5.5 (4.4–7.0)	5.8 (4.5–7.0)	0.605
ASDAS-ESR	3.3 (2.9–3.5)	3.2 (2.2–3.5)	0.242
BASFI	5.8 (3.6–7.2)	5.3 (4.3–6.8)	0.972
BASMI	5.0 (3.0–6.0)	4.5 (3.0–8.0)	0.458
MASES	2.0 (0.0–5.0)	0.0 (0.0–5.2)	0.498
Laboratory findings			
CRP, mg/L	10.0 (3.0–21.0)	11.8 (5.8–20.0)	0.852
ESR, mm/h	17.0 (8.0–27.0)	21.0 (4.8–38.8)	0.648
Fecal calprotectin, µg/g	38.3 (23.3–61.6), *n* = 34	67.7 (39.1–154.0), *n* = 5	0.284
Comorbidities and extra-musculoskeletal manifestations			
Osteoarthritis, *n*/N (%)	17/84 (20.2)	10/21 (47.6)	0.023
Arterial hypertension, *n* (%)	21/89 (23.6)	6/24 (25.0)	1.000
Osteoporosis, *n*/N (%)	15/85 (17.6)	4/24 (16.7)	1.000
Uveitis, *n*/N (%)	6/71 (8.5)	2/22 (9.1)	1.000
Inflammatory bowel disease, *n*/N (%)	8/88 (9.1)	1/24 (4.2)	0.681
Psoriasis, *n*/N (%)	8/89 (9.0)	3/24 (12.5)	0.698

Note. Values are median (25th–75th percentile) unless otherwise indicated. Percentages are calculated using available observations; denominators are shown where data were missing. Continuous variables were compared using the Wilcoxon rank-sum test and categorical variables using Fisher’s exact test. All comparisons were exploratory and unadjusted; no adjustment for multiple comparisons was performed. Abbreviations: ASDAS-ESR, Ankylosing Spondylitis Disease Activity Score based on erythrocyte sedimentation rate; BASDAI, Bath Ankylosing Spondylitis Disease Activity Index; BASFI, Bath Ankylosing Spondylitis Functional Index; BASMI, Bath Ankylosing Spondylitis Metrology Index; CRP, C-reactive protein; ESR, erythrocyte sedimentation rate; MASES, Maastricht Ankylosing Spondylitis Enthesitis Score. Data are presented using available-case denominators. Denominators may vary between variables because of missing baseline data; no imputation was performed for missing baseline values.

**Table 3:** Cox proportional hazards analysis of factors associated with confirmed golimumab discontinuation.

Variable	Univariable HR (95% CI)	*p*-Value	Adjusted HR (95% CI)	*p*-Value
RDCI total score, per 1-point increase	1.357 (1.085–1.696)	0.007	1.312 (1.042–1.653)	0.021
Functional class III–IV vs. I–II	3.641 (1.590–8.334)	0.002	2.412 (1.037–5.611)	0.041
Previous csDMARD history		0.024 *		0.086 *
Sulfasalazine vs. none	3.022 (0.898–10.175)	0.074	2.769 (0.817–9.388)	0.102
Methotrexate vs. none	0.546 (0.057–5.253)	0.601	0.729 (0.074–7.132)	0.785

* Overall likelihood-ratio test for the three-level categorical variable. The multivariable model included 113 patients and 24 confirmed discontinuation events. Functional class I–II and no previous csDMARD use were used as the reference categories. The proportional hazard assumption was not violated (global Schoenfeld test, *p* = 0.82).

**Table 4:** Clinical outcomes during prospective follow-up among patients with available on-treatment assessments.

Outcome	Baseline (*n* = 113)	Week 12 (*n* = 113)	Week 24 (*n* = 107)	Week 52 (*n* = 105)	Week 104 (*n* = 99)	Week 144 (*n* = 88)	Paired Change at Week 144 *	*p*-Value *
BASDAI	5.5 (4.4–7.0)	4.0 (3.5–5.1)	3.6 (3.0–4.0)	2.5 (2.0–3.6)	2.1 (1.9–3.35)	2.0 (1.8–2.43)	−3.35 (−5.00 to −2.38)	<0.001
ASDAS-ESR	3.2 (2.8–3.5)	2.1 (2.0–3.0)	2.0 (1.75–2.1)	1.8 (1.2–2.0)	1.4 (1.0–1.9)	1.0 (0.7–1.2)	−2.20 (−2.50 to −1.90)	<0.001
BASFI	5.7 (3.9–7.2)	5.0 (3.3–6.0)	4.0 (3.0–5.0)	3.7 (2.0–4.0)	3.0 (2.0–4.0)	3.0 (2.0–3.8)	−2.65 (−4.13 to −1.20)	<0.001
BASMI	5.0 (3.0–7.0)	4.0 (3.0–6.0)	4.0 (2.0–5.5)	4.0 (2.0–5.0)	4.0 (2.0–5.0)	3.8 (1.0–5.0)	−1.00 (−2.00 to −0.08)	<0.001
MASES	2.0 (0.0–5.0)	0.0 (0.0–3.0)	0.0 (0.0–1.0)	0.0 (0.0–0.0)	0.0 (0.0–0.0)	0.0 (0.0–0.0)	−2.00 (−5.00 to 0.00)	<0.001

Values are median (interquartile range). * Baseline values are reported for the entire cohort (*n* = 113), whereas paired baseline-to-week 144 analyses were performed among the 88 patients with valid on-treatment assessments at both time points. Therefore, baseline medians in the paired subgroup may differ from those reported for the full cohort. Changes were calculated as week 144 minus baseline and are presented as median (interquartile range). *p*-values were obtained using the paired Wilcoxon signed-rank test. Post-discontinuation measurements were excluded.

**Table 5:** Reasons for confirmed permanent golimumab discontinuation (*n* = 24).

Reason for Discontinuation	*n*	% *
Loss of effectiveness	13	54.2
Confirmed tuberculosis	5	20.8
Other infectious complications ^†^	3	12.5
Psycho-emotional/depressive symptoms	1	4.2
Malignancy ^‡^	1	4.2
Allergic reaction ^§^	1	4.2
Total	24	100.0

* Percentages were calculated among patients with confirmed permanent golimumab discontinuation (*n* = 24). ^†^ Other infectious complications comprised two documented cases of COVID-19 and one case of hepatitis C. ^‡^ Malignancy was a stage II right renal cancer (T2bNxM0). ^§^ The allergic reaction was angioedema (Quincke’s edema).

## Data Availability

The data supporting the findings of this study are available from the corresponding author upon reasonable request. The data are not publicly available due to privacy and ethical restrictions related to patient data.

## Supplementary Materials

The following supporting information can be downloaded at https://www.mdpi.com/article/10.3390/jcm15208099/s1, Table S1: STROBE checklist for cohort studies.

## Abbreviations

The following abbreviations are used in this manuscript:

ASDAS-ESR	Ankylosing Spondylitis Disease Activity Score using erythrocyte sedimentation rate
axSpA	Axial spondyloarthritis
BASDAI	Bath Ankylosing Spondylitis Disease Activity Index
BASFI	Bath Ankylosing Spondylitis Functional Index
BASMI	Bath Ankylosing Spondylitis Metrology Index
bDMARD	Biologic disease-modifying antirheumatic drug
CI	Confidence interval
CRP	C-reactive protein
csDMARD	Conventional synthetic disease-modifying antirheumatic drug
DMARD	Disease-modifying antirheumatic drug
ESR	Erythrocyte sedimentation rate
EULAR	European Alliance of Associations for Rheumatology
HLA-B27	Human leukocyte antigen B27
HR	Hazard ratio
MASES	Maastricht Ankylosing Spondylitis Enthesitis Score
RDCI	Rheumatic Disease Comorbidity Index
TNFi	Tumor necrosis factor inhibitor
