# Supplementary material for: Long-Term Golimumab Treatment Persistence and Clinical Outcomes in Axial Spondyloarthritis: A Real-World Cohort from Kazakhstan
Source: J Clin Med. 2026 Oct 19;15(20):8099. doi: 10.3390/jcm15208099 (PMC13706086; doi:10.3390/jcm15208099)
Supplement: Supplementary file 1 [file jcm-15-08099-s001.zip › jcm-4551847-supplementary.pdf]

Table S1. STROBE Statement—Checklist of items that should be included in reports of cohort studies

Manuscript: Long-term golimumab treatment persistence and clinical outcomes in axial spondyloarthritis: a real-world cohort from Kazakhstan

Item	No.	STROBE recommendation	Where reported in manuscript
Title and abstract	1a	Indicate the study's design with a commonly used term in the title or the abstract.	pp. 1–2 (title; Abstract, Methods)
Title and abstract	1b	Provide in the abstract an informative and balanced summary of what was done and what was found.	p. 2 (Abstract)
Background/rationale	2	Explain the scientific background and rationale for the investigation being reported.	p. 3 (Introduction)
Objectives	3	State specific objectives, including any prespecified hypotheses.	p. 3 (end of Introduction)
Study design	4	Present key elements of study design early in the paper.	p. 3 (Section 2.1)
Setting	5	Describe the setting, locations, and relevant dates, including periods of recruitment, exposure, follow-up, and data collection.	pp. 3–4 (Sections 2.1–2.2)
Participants	6a	Give the eligibility criteria, and the sources and methods of selection of participants. Describe methods of follow-up.	pp. 3–4 (Sections 2.1–2.2); p. 8 (Figure 1)
Participants	6b	For matched studies, give matching criteria and number of exposed and unexposed.	Not applicable (unmatched single-cohort study)
Variables	7	Clearly define all outcomes, exposures, predictors, potential confounders, and effect modifiers. Give diagnostic criteria, if applicable.	pp. 4–6 (Sections 2.2–2.5)
Data sources/measurement	8	For each variable of interest, give sources of data and details of methods of assessment (measurement). Describe comparability of assessment methods if there is more than one group.	pp. 4–6 (Sections 2.3–2.5)
Bias	9	Describe any efforts to address potential sources of bias.	pp. 5–7 (on-treatment assessments, NRI, censoring, restricted Cox model); pp. 20–21 (Section 4.2)
Study size	10	Explain how the study size was arrived at.	p. 4 (Section 2.2: consecutive eligibility assessment); p. 8 (Figure 1: 140 assessed, 113 included)
Quantitative variables	11	Explain how quantitative variables were handled in the analyses. If applicable, describe which groupings were chosen and why.	pp. 5–7 (Sections 2.3 and 2.7; functional class dichotomization and statistical handling)
Statistical methods	12a	Describe all statistical methods, including those used to control for confounding.	pp. 6–7 (Section 2.7)
Statistical methods	12b	Describe any methods used to examine subgroups and interactions.	p. 7 (Section 2.7: subgroup comparisons using the log-rank test; no interaction analyses performed).
Statistical methods	12c	Explain how missing data were addressed.	p. 6 (Section 2.7: available-case denominators, no baseline imputation, NRI for responder analyses)
Statistical methods	12d	If applicable, explain how loss to follow-up was addressed.	pp. 5–7 (Sections 2.4 and 2.7: censoring at last documented follow-up; NRI for responder analyses)
Statistical methods	12e	Describe any sensitivity analyses.	p. 7 (Section 2.7: restricted discontinuation endpoint)
Participants	13a	Report numbers of individuals at each stage of study—e.g., numbers potentially eligible, examined for eligibility, confirmed eligible, included in the study, completing follow-up, and analysed.	p. 8 (Section 3.1 and Figure 1); pp. 13–17 (analysis denominators)
Participants	13b	Give reasons for non-participation at each stage.	p. 8 (Section 3.1 and Figure 1)

Item	No.	STROBE recommendation	Where reported in manuscript
Participants	13c	Consider use of a flow diagram.	p. 8 (Figure 1)
Descriptive data	14a	Give characteristics of study participants and information on exposures and potential confounders.	pp. 8–10 (Section 3.1; Tables 1–2)
Descriptive data	14b	Indicate number of participants with missing data for each variable of interest.	pp. 9–10 (Tables 1–2; available-case denominators)
Descriptive data	14c	Summarise follow-up time (e.g., average and total amount).	p. 11 (Section 3.2: reverse Kaplan–Meier median follow-up, 36 months)
Outcome data	15	Report numbers of outcome events or summary measures over time.	pp. 11–17 (Sections 3.2–3.6; Tables 3–5; Figures 2–5)
Main results	16a	Give unadjusted estimates and, if applicable, confounder-adjusted estimates and their precision (e.g., 95% confidence interval). Make clear which confounders were adjusted for and why they were included.	pp. 12–13 (Section 3.3; Table 3; Figure 3); pp. 6–7 (model specification and covariate-selection rationale)
Main results	16b	Report category boundaries when continuous variables were categorized.	pp. 5–7 and 12–13 (functional class III–IV vs. I–II; reference categories stated)
Main results	16c	If relevant, consider translating estimates of relative risk into absolute risk for a meaningful time period.	p. 11 (Section 3.2: absolute treatment-persistence probabilities at 6, 12, 24, and 36 months).
Other analyses	17	Report other analyses done—e.g., analyses of subgroups and interactions, and sensitivity analyses.	pp. 16–17 (Sections 3.5–3.6: NRI responder analyses and sensitivity analysis using a restricted discontinuation endpoint).
Key results	18	Summarise key results with reference to study objectives.	pp. 18–20 (Section 4, Discussion)
Limitations	19	Discuss limitations of the study, taking into account sources of potential bias or imprecision. Discuss both direction and magnitude of any potential bias.	pp. 20–21 (Section 4.2, Strengths and limitations)
Interpretation	20	Give a cautious overall interpretation of results considering objectives, limitations, multiplicity of analyses, results from similar studies, and other relevant evidence.	pp. 18–21 (Discussion; Section 4.2)
Generalisability	21	Discuss the generalisability (external validity) of the study results.	pp. 20–21 (Section 4.2: two-center Almaty cohort and limits to wider Kazakhstan population)
Funding	22	Give the source of funding and the role of the funders for the present study and, if applicable, for the original study on which the present article is based.	p. 21 (Funding: no external funding)

* STROBE items 8, 13, 14, and 15 recommend reporting information separately for exposed and unexposed groups when applicable. This study was a single golimumab-treated cohort; comparisons by confirmed discontinuation status are presented in Tables 1–2 where relevant.

Abbreviations: NRI, non-responder imputation; STROBE, Strengthening the Reporting of Observational Studies in Epidemiology.
